# Ten simple rules for responsible use of AI and LLMs in computational biology

**DOI:** 10.1371/journal.pcbi.1014882

**Published:** 2026-10-22

**Authors:** Noam Auslander

**Affiliations:** Program in Molecular and Cellular Oncogenesis, The Wistar Institute, Philadelphia, Pennsylvania, United States of America; Carnegie Mellon University, UNITED STATES OF AMERICA

## Introduction

Artificial intelligence (AI) and large language models (LLMs) are rapidly transforming the practice of computational biology, offering faster outcomes, new ways to generate hypotheses, and accelerated access for analyzing complex datasets and identifying potential solutions. Like many computational disciplines, computational biology has rapidly adopted AI, which has achieved state of the art performance in diverse tasks ranging from protein structure prediction [1,2] and new sequence generation [3–5] to medical applications and patient interactions [6,7]. However, computational biology presents a distinct challenge because AI-generated outputs can directly become analytical decisions, computational workflows, and biological interpretations that ultimately determine scientific conclusions [8–13].

The growing accessibility and hype surrounding LLMs have lowered barriers to entry, enabling researchers with limited computational training, including wet-lab biologists and clinicians, to adopt advanced computational methods, whose outputs can directly inform biological conclusions and clinical decisions. Because these systems can produce reasonable-appearing but incorrect results that obscure the provenance of information, uncritical or unsupervised use can undermine reproducibility, interpretability, and scientific rigor, leading to misleading conclusions or irreproducible results [14–17]. The inherently interdisciplinary nature of computational biology, which brings together researchers with diverse expertise and widely varying levels of computational knowledge, further compounds these challenges and introduces new ones unique to the field.

The rapid integration of LLM chatbots into scientific workflows has prompted emerging perspectives outlining use cases for their application in computational biology and the broader scientific landscape [18–23]. These guidelines largely provide recommendations for responsible adoption by describing appropriate use and scientific best practices across diverse scientific activities, including computational biology [18], grant writing [23], and broader scientific workflows [19–22]. While all these perspectives acknowledge potential risks and inappropriate uses, none explicitly define the boundaries between appropriate assistance and problematic delegation in computational biology, or address the challenges that arise when AI-generated outputs can directly shape scientific reasoning and conclusions.

The absence of clear boundaries is not incidental, it reflects a unique gray zone in computational biology. Unlike experimental disciplines, where inappropriate use of AI, such as generating microscopy images or modifying PCR plots, is widely recognized as unacceptable, AI-generated code, analyses, and visualization in computational research may directly and appropriately contribute to publishable outputs. This dual role, as both supporting tools and generators of scientific content, obscures the boundary between acceptable and problematic use. This perspective provides practical guidance for researchers developing or applying computational methods, helping them navigate both the opportunities and the risks of AI-assisted bioinformatic research.

## 1. Do not use generative AI to create, modify, or cosmetically enhance primary data, figures, or analytical outputs

Generative AI systems are prone to hallucinations [24] and may produce outputs that reflect user expectations [25,26] rather than underlying data. They should not be used to generate or modify raw data or produce presentation- or publication-ready figures. A key purpose of computational analysis pipelines is to ensure that results are directly and reproducibly derived from raw data without subjective alteration. Using generative AI to produce or modify scientific data or figures risks introducing undetectable errors and undermines scientific integrity. All data-processing and visualization should be conducted through auditable, deterministic workflows to preserve transparency and reproducibility, for example, using Python or R scripts maintained in GitHub. Moreover, because chatbot outputs are highly responsive to user prompts, interactions can intentionally or unintentionally manipulate scientific results in ways that will be difficult to audit. Examples of problematic prompts include those aimed at enhancing or altering the appearance of data, such as “how can these curves look more different”, “can you remove outliers to make the trend clearer”, or “how can I smooth these plots to make the trend more convincing”. Such prompts must be explicitly prohibited. Even seemingly innocuous prompts may inadvertently encourage problematic manipulation of scientific data. Examples include requests to rescale axes, recolor visualizations, change normalization, or combine samples to emphasize presented scientific patterns. Generative AI should therefore be strictly limited to supporting tasks, such as refining or debugging visualization code [18], and never used directly to produce report-ready plots.

## 2. Do not delegate statistical analysis or statistical test selection to LLMs

LLMs should not be used to generate quantitative statistical results (e.g., p-values, confidence intervals, coefficients, or model predictions), as their outputs may be incorrect, nonreproducible, or based on flawed assumptions. However, these tools may be useful for explaining commonly used statistical methods and the situations in which they are typically applied. They may also suggest broader analytical strategies and statistical approaches for consideration, provided that these suggestions are independently evaluated against established statistical resources. The final choice of statistical test for a specific hypothesis, however, should remain the responsibility of the researcher and predefined before data analysis. Using LLMs for statistical test selection substantially increases the risk of p-hacking by lowering the technical barrier to rapidly exploring alternative analyses, models, transformations, and outcomes until a significant result is obtained. This overlaps with well-known p-hacking practices, including selective reporting of outcomes or predictors, outlier exclusion, variable transformation or discretization, scale redefinition, and model parameterization [27].

## 3. Ensure adequate expertise before using generative AI for coding

Generative AI can accelerate code development for users with sufficient expertise. These tools can help draft code skeletons, suggest relevant packages, and identify or correct bugs, often more efficiently than searching across forums. They can also assist with documentation and streamline data-processing scripts, as discussed more comprehensively in [18].

However, their use should be restricted to users who can understand the task, critically assess the proposed solution, and verify its validity [18]. While fluency in the programming language and conceptual understanding of the task are widely considered essential [20], the criteria for defining these competencies remain insufficiently defined.

I propose a guiding principle for the use of LLM-generated code in research: users should be capable of independently developing the underlying algorithmic solution without AI assistance. In such cases, LLM-generated code serves primarily to improve efficiency. AI may assist with technical implementation, such as optimization, parallelization, or software packaging, provided that the user can verify their correctness, for example, by testing against a reference implementation or testing with benchmark inputs. If the user has not developed the necessary understanding and problem-solving skills to address the underlying problem, however, gains in speed may come at the expense of correctness and reliability.

This principle enables a key sanity check. The core logic of LLM-generated code should reflect constructs the user could have derived independently, even if the implementation is technically more sophisticated than the user could produce. Responsibility for code validity rests with the researcher, and LLM-generated code warrants at least as much scrutiny as one’s own. This principle does not preclude the use of LLMs to support learning, but rather it defines when LLM-generated code could be incorporated into research. Researchers may use LLMs to learn new computational approaches but should independently understand and master the underlying solution before relying on LLM-generated code in their research.

## 4. Take full responsibility for every line of AI-generated code. Do not vibe-code science

“Vibing” code, pasting subjective descriptions into an LLM, accepting the output with minimal scrutiny, and running it without deep understanding of the underlying logic, assumptions, numerical stability, edge cases, or statistical validity, should never be acceptable in science. It fundamentally risks irreproducible, biased, incorrect, or outright fabricated results in computational biology and related fields. Code that “feels right” or produces superficially nice plots is not science, and it substitutes aesthetic persuasion for empirical integrity.

It is sometimes argued that trainees should be allowed to use generative AI in research because it is a common practice in industry. There are at least two concerns with that argument. First, it is potentially detrimental to learning. Because generative AI has only recently been incorporated into software development, the vast majority of today’s industry professionals were trained without it and therefore possess the foundational skills needed to use it appropriately and to critically judge its output. However, allowing trainees to use generative AI for coding may hinder the development of foundational skills [28,29]. Second, science and industry have fundamentally different goals when using code [30,31]. While code that runs successfully is sufficient for many industry applications, reaching the correct scientific conclusions is the main goal of academic research. I have seen more bioinformatic packages that fail to run than ones that run smoothly on first application, yet associated papers continue to be widely cited. Poorly written code is frustrating, but code generated without careful supervision and a thorough understanding can produce incorrect results and lead to erroneous scientific conclusions, which may be more problematic in research [32].

Therefore, researchers should treat LLM-generated code as a contribution from an anonymous user, or inspiration at best. To take ownership of the resulting scientific conclusions, researchers must independently verify every line, understand the algorithm, check mathematical correctness, and test using reconstructed input-output pairs and benchmarks. Scientific code must properly handle domain-specific challenges such as missing data, low coverage, insufficient sequencing depth, batch effects, multiple-testing correction, and outliers. Always verify units, scaling, and normalization appropriate to the biological or computational problem at hand. Results should ultimately be reproduced using version-controlled, human-written (or heavily audited) code, and final package outputs should ideally be independently reviewed by other researchers. Science requires mechanistic comprehension and reproducible rigor, not superficial confidence derived from the chatbot “vibes”.

## 5. Use generative AI to write more unit tests, validation checks, and identify bugs in your code

Generative AI can serve as an additional code reviewer by identifying potential bugs, suggesting edge cases and failure modes, and recommending improvements to code organization, documentation, and package usability. In computational biology, it can also help generate tests for common domain-specific failure modes, such as incorrect gene identifier conversions, sample-label mismatches, missing metadata, reference genome inconsistencies, sparse matrices, or malformed sequencing files. It can rapidly generate unit tests, input validation procedures, and assertions that improve software reliability, reproducibility, and user-friendliness. These capabilities can increase test coverage and help uncover errors before they affect downstream biological interpretation, particularly when expert code review is limited. However, AI-generated tests and bug reports should not be assumed to be correct or comprehensive. The same principles of independent verification and careful code review of rules 3 and 4 apply to AI-generated tests and AI-suggested bug fixes.

## 6. Use AI chatbots to recall syntax, not scientific reasoning

Many computational biologists routinely switch between programming languages and software environments. For example, they may move from Python to Bash for command-line workflows or from Python to R for data visualization, where language-specific syntax can become increasingly cumbersome, particularly when using libraries such as Matplotlib or ggplot2. In these cases, the researcher already understands the algorithmic, statistical, or biological logic of the intended analysis. What is missing is fluency in the required syntax to implement it correctly. Seeing the correct syntax is often sufficient to quickly produce an accurate implementation, and AI chatbots can retrieve that syntax much more quickly than traditional documentation or web searches. This distinction suggests another practical test for appropriate AI use. If a researcher can independently verify that AI-generated output correctly implements a decision they have already made, the tool is serving as an accelerator. If they cannot evaluate the output against their expectation because the underlying decision was outsourced, the use becomes delegation rather than assistance.

## 7. Use LLMs for preliminary exploration of data patterns, but validate findings through appropriate, reproducible analyses

LLMs can help identify potential patterns, provide clues to generate hypotheses, and suggest avenues for exploration in datasets given a set of results. However, it is important that this application be restricted to de-identified and public-domain data. For example, generative AI can highlight recurring features that may help determine whether a dataset contains patterns warranting formal follow-up analysis by answering prompts, such as “what enzymatic reactions are over-represented among these metabolites”, “what functions are shared across these proteins” or “what are the shared properties among these amino acids”. They can also compare entities with heterogeneous naming conventions, such as drug names, model organism nomenclature, transcript identifiers, or gene aliases, helping identify potential matches or relationships. However, generative AI-suggested insights are inherently preliminary and must be validated against the primary literature, curated databases or rigorous quantitative, statistical, computational, or experimental analysis. Consistent with rules 1 and 2, LLMs should not be used to generate quantitative measures, statistical outputs, or figures during this exploratory process. Treat generative AI outputs as tools that provide directions for follow-up, rather than as evidence or definitive conclusions.

## 8. Employ extreme caution with clinical and identifiable data

Medical, clinical, and most sequencing datasets contain sensitive, proprietary, or personally identifiable information subject to strict legal and ethical sharing restrictions. Submitting such data to open-source, public, or third-party LLM services risks permanent storage, use in model training, memorization, and security breaches. These exposures can seriously endanger patient privacy, intellectual property, biosecurity, and competitive or collaborative advantages.

While these concerns have been noted [20], what constitutes a breach is often not well defined in practice. To provide actionable guidance, LLM use can be categorized into three groups: (1) “Protect” - Do not input raw sequence, sensitive, confidential, personally identifiable, clinical, or proprietary data into public or third-party LLMs unless their use is explicitly authorized under institutional policies and data protection requirements [33]. (2) “Obtain permissions“- Before using LLMs with drafts, unpublished results, code, ideas, algorithms, or other nonpublic research material, ensure that you have the necessary authorization from collaborators, institutions, or other relevant parties [34]. (3) “Use responsibly“- Use LLMs for low-risk tasks that do not involve sharing usable private information, such as identifying software packages, querying terminology, debugging isolated commands, or other minimal, nonsensitive interactions. As a guiding principle, all inputs should be treated as potentially persistent and shareable, ensuring adherence to ethical, legal, and scientific standards.

## 9. Keep scientific judgment under human control, especially when using agentic AI

The incorporation of agentic AI into scientific research is accelerating [35], with end-to-end AI-driven scientific workflows increasingly being proposed [36,37]. Although these systems cannot yet perform wet-lab experiments autonomously, they are increasingly capable of carrying out many computational aspects of biomedical research, including literature review, hypothesis generation, code development, data analysis, manuscript drafting, and guiding researchers throughout the scientific process. This growing capability raises a responsibility that extends beyond the scientific validity of a researcher’s own work to its impact on the broader scientific community. AI can substantially lower the barrier to contributing to scientific software, and can allow inexperienced users to make contributions without engaging with the community, consuming reviewer time and resources without necessarily improving the software [38]. When used with overly permissive settings or insufficient oversight, agentic AI systems may generate outputs that inadvertently violate software licenses, reuse copyrighted code, or irreversibly modify or delete valuable research data [39–42], exposing researchers and institutions to legal, ethical, and scientific risks.

## 10. Use LLMs to improve manuscript consistency after exhausting human review

Scientific manuscripts, particularly those describing computational studies, undergo repeated revisions that can introduce inconsistencies between the main text, supplementary materials, figures, code, and analysis workflows. Even careful authors may overlook such discrepancies after multiple revision cycles. LLMs can assist by identifying potential inconsistencies, for example by comparing methods described in the main and supplementary text, checking whether code matches methodological descriptions, or verifying consistency between figures, tables, and results. However, LLMs should complement, rather than replace, careful human review, and every suggested correction should be independently verified before incorporation.

## References

[1] Jumper J, Evans R, Pritzel A, Green T, Figurnov M, Ronneberger O, et al. Highly accurate protein structure prediction with AlphaFold. Nature. 2021;596(7873):583–9. doi: 10.1038/s41586-021-03819-2 34265844 PMC8371605

[2] Abramson J, Adler J, Dunger J, Evans R, Green T, Pritzel A, et al. Accurate structure prediction of biomolecular interactions with AlphaFold 3. Nature. 2024;630(8016):493–500. doi: 10.1038/s41586-024-07487-w 38718835 PMC11168924

[3] Lin Z, Akin H, Rao R, Hie B, Zhu Z, Lu W, et al. Evolutionary-scale prediction of atomic-level protein structure with a language model. Science. 2023;379(6637):1123–30. doi: 10.1126/science.ade2574 36927031

[4] Nijkamp E, Ruffolo JA, Weinstein EN, Naik N, Madani A. ProGen2: exploring the boundaries of protein language models. Cell Syst. 2023;14(11):968-978.e3. doi: 10.1016/j.cels.2023.10.002 37909046

[5] Ferruz N, Schmidt S, Höcker B. ProtGPT2 is a deep unsupervised language model for protein design. Nat Commun. 2022;13(1):4348. doi: 10.1038/s41467-022-32007-7 35896542 PMC9329459

[6] Ayers JW, Poliak A, Dredze M, Leas EC, Zhu Z, Kelley JB, et al. Comparing physician and artificial intelligence chatbot responses to patient questions posted to a public social media forum. JAMA Intern Med. 2023;183(6):589–96. doi: 10.1001/jamainternmed.2023.1838 37115527 PMC10148230

[7] Sarraju A, Bruemmer D, Van Iterson E, Cho L, Rodriguez F, Laffin L. Appropriateness of cardiovascular disease prevention recommendations obtained from a popular online chat-based artificial intelligence model. JAMA. 2023;329: 842–4. doi: 10.1001/jama.2023.1044 PMC10015303 36735264

[8] Simon E, Swanson K, Zou J. Language models for biological research: a primer. Nat Methods. 2024;21(8):1422–9. doi: 10.1038/s41592-024-02354-y 39122951

[9] Swanson K, Wu W, Bulaong NL, Pak JE, Zou J. The virtual lab of AI agents designs new SARS-CoV-2 nanobodies. Nature. 2025;646(8085):716–23. doi: 10.1038/s41586-025-09442-9 40730228

[10] Schaefer M, Peneder P, Malzl D, Lombardo SD, Peycheva M, Burton J, et al. Multimodal learning enables chat-based exploration of single-cell data. Nat Biotechnol. 2026;44(10):1722–32. doi: 10.1038/s41587-025-02857-9 41219484 PMC13654808

[11] Bioinformatics and biomedical informatics with ChatGPT: Year one review - Wang; 2024 - Quantitative Biology - Wiley Online Library. [cited 6 Aug 2026]. Available from: https://onlinelibrary.wiley.com/doi/full/10.1002/qub2.67 10.1002/qub2.67 PMC11446534 39364207

[12] Wang Z, Jin Q, Wei C-H, Tian S, Lai P-T, Zhu Q, et al. GeneAgent: self-verification language agent for gene-set analysis using domain databases. Nat Methods. 2025;22(8):1677–85. doi: 10.1038/s41592-025-02748-6 40721871 PMC12328209

[13] Mehandru N, Hall AK, Melnichenko O, Dubinina Y, Tsirulnikov D, Bamman D, et al. BioAgents: bridging the gap in bioinformatics analysis with multi-agent systems. Sci Rep. 2025;15(1):39036. doi: 10.1038/s41598-025-25919-z 41203737 PMC12594986

[14] Gravel J, D’Amours-Gravel M, Osmanlliu E. Learning to fake it: limited responses and fabricated references provided by ChatGPT for medical questions. Mayo Clin Proc Digit Health. 2023;1(3):226–34. doi: 10.1016/j.mcpdig.2023.05.004 40206627 PMC11975740

[15] Farquhar S, Kossen J, Kuhn L, Gal Y. Detecting hallucinations in large language models using semantic entropy. Nature. 2024;630(8017):625–30. doi: 10.1038/s41586-024-07421-0 38898292 PMC11186750

[16] Zhao Z, Wang Y, Stuart T, Vaan MD, Ginsparg P, Yin Y. LLM hallucinations in the wild: Large-scale evidence from non-existent citations. arXiv. 2026. doi: 10.48550/arXiv.2605.07723

[17] Chen S, Gao M, Sasse K, Hartvigsen T, Anthony B, Fan L, et al. When helpfulness backfires: LLMs and the risk of false medical information due to sycophantic behavior. NPJ Digit Med. 2025;8(1):605. doi: 10.1038/s41746-025-02008-z 41107408 PMC12534679

[18] Lubiana T, Lopes R, Medeiros P, Silva JC, Goncalves ANA, Maracaja-Coutinho V, et al. Ten quick tips for harnessing the power of ChatGPT in computational biology. PLoS Comput Biol. 2023;19(8):e1011319. doi: 10.1371/journal.pcbi.1011319 37561669 PMC10414555

[19] Stokel-Walker C, Van Noorden R. What ChatGPT and generative AI mean for science. Nature. 2023;614(7947):214–6. doi: 10.1038/d41586-023-00340-6 36747115

[20] Smith GR, Bello C, Bialic-Murphy L, Clark E, Delavaux CS, Fournier de Lauriere C, et al. Ten simple rules for using large language models in science, version 1.0. PLoS Comput Biol. 2024;20(1):e1011767. doi: 10.1371/journal.pcbi.1011767 38295118 PMC10829980

[21] Helmy M, Jin L, Alhossary A, Mansour T, Pellagrina D, Selvarajoo K. Ten simple rules for optimal and careful use of generative AI in science. PLoS Comput Biol. 2025;21(10):e1013588. doi: 10.1371/journal.pcbi.1013588 41150680 PMC12561928

[22] Crilly A, Malivert A, Jørgensen ACS, Heaney CE, Vera Gonzalez GI, Ghosh M, et al. Ten simple rules for navigating AI in science. PLoS Comput Biol. 2025;21(7):e1013259. doi: 10.1371/journal.pcbi.1013259 40680100 PMC12273916

[23] Seckel E, Stephens BY, Rodriguez F. Ten simple rules to leverage large language models for getting grants. PLoS Comput Biol. 2024;20(3):e1011863. doi: 10.1371/journal.pcbi.1011863 38427611 PMC10906892

[24] Huang L, Yu W, Ma W, Zhong W, Feng Z, Wang H, et al. A survey on hallucination in large language models: principles, taxonomy, challenges, and open questions. ACM Trans Inf Syst. 2025;43:42:1-42:55. doi: 10.1145/3703155

[25] Kang WC, Ni J, Mehta N, Sathiamoorthy M, Hong L, Chi E. Do LLMs understand user preferences? Evaluating LLMs on user rating prediction. arXiv. 2023. doi: 10.48550/arXiv.2305.06474

[26] Jiang B, Hao Z, Cho YM, Li B, Yuan Y, Chen S, et al. Know me, respond to me: benchmarking LLMs for dynamic user profiling and personalized responses at scale. arXiv. 2025. doi: 10.48550/arXiv.2504.14225

[27] Big little lies: a compendium and simulation of p-hacking strategies. Royal Society Open Science. The Royal Society. 2026. [cited 30 July 2026]. Available from: https://royalsocietypublishing.org/rsos/article/10/2/220346/92017/Big-little-lies-a-compendium-and-simulation-of-p 10.1098/rsos.220346 PMC9905987 36778954

[28] Literature review on the integration of generative AI in programming education. In: International Journal of Artificial Intelligence in Education. Springer Nature Link. 2025 [cited 30 July 2026]. Available from: https://link.springer.com/article/10.1007/s40593-025-00524-3

[29] Systematic review of large language model applications in programming education; 2025 [cited 30 July 2026]. Available from: https://journals.sagepub.com/doi/full/10.3233/FAIA250512

[30] Re-run, repeat, reproduce, reuse, replicate: transforming code into scientific contributions. Frontiers. Available from: https://www.frontiersin.org/journals/neuroinformatics/articles/10.3389/fninf.2017.00069/full. Accessed 2026 July 30. 10.3389/fninf.2017.00069 PMC5758530 29354046

[31] Reproducibility and replicability in science. Available from: https://www.nationalacademies.org/projects/DBASSE-BBCSS-17-03/publication/25303. Accessed 2026 July 30.

[32] Producing wrong data without doing anything obviously wrong! ACM SIGPLAN Notices. 2026 [cited 30 July 2026]. Available from: https://dl.acm.org/doi/abs/10.1145/1508284.1508275

[33] Ross R, Pillitteri V. Protecting controlled unclassified information in nonfederal systems and organizations. NIST Special Publication (SP) 800-171 Rev. 3. National Institute of Standards and Technology; 2024. doi: 10.6028/NIST.SP.800-171r3

[34] CDC. Considerations for disclosing generative AI use in scientific work. Artificial Intelligence. 2026. Available from: https://www.cdc.gov/ai/resources/considerations-for-generative-ai-use-in-scientific-work.html. Accessed 2026 July 31.

[35] Gridach M, Nanavati J, Mack C, Abidine KZE, Mendes L. Agentic AI for scientific discovery: a survey of progress, challenges, and future directions. 2025. Available from: https://openreview.net/forum?id=TyCYakX9BD

[36] Lu C, Lu C, Lange RT, Yamada Y, Hu S, Foerster J, et al. Towards end-to-end automation of AI research. Nature. 2026;651(8107):914–9. doi: 10.1038/s41586-026-10265-5 41882133 PMC13017497

[37] Ghareeb AE, Chang B, Mitchener L, Yiu A, Szostkiewicz CJ, Shved D, et al. A multi-agent system for automating scientific discovery. Nature. 2026;655(8122):497–505. doi: 10.1038/s41586-026-10652-y 42156546 PMC13346116

[38] Ogenrwot D, Businge J. How AI coding agents modify code: a large-scale study of GitHub pull requests. In: Proceedings of the 23rd International Conference on Mining Software Repositories; 2026. pp. 924–8. 10.1145/3793302.3793603

[39] Colombo G, Mariani L, Micucci D, Riganelli O. On the possibility of breaking copyleft licenses when reusing code generated by ChatGPT. arXiv. 2025. doi: 10.48550/arXiv.2502.05023

[40] A first look at license compliance capability of LLMs in code generation. 2026 [cited 31 July 2026]. Available: https://arxiv.org/html/2408.02487v1

[41] Zhong SW, Liao J, Liu J, Zheng M, Arpaci-Dusseau AC, Arpaci-Dusseau RH. Don’t let AI agents YOLO your files: shifting information and control to filesystems for agent safety and autonomy. arXiv. 2026. doi: 10.48550/arXiv.2604.13536

[42] Mansoor S. Claude-powered AI agent’s confession after deleting a firm’s entire database: ‘I violated every principle I was given’. The Guardian. 2026. Available from: https://www.theguardian.com/technology/2026/apr/29/claude-ai-deletes-firm-database Accessed 31 July 2026.

